# Maximum Measurement Range and Accuracy of SAW Reflective Delay Line Sensors

**DOI:** 10.3390/s151026643

**Published:** 2015-10-20

**Authors:** Zehua Zheng, Tao Han, Peng Qin

**Affiliations:** Department of Instrument Science and Engineering, Shanghai Jiao Tong University, Shanghai 200240, China; E-Mails: sjtuzzh@163.com (Z.Z.); pqin@sjtu.edu.cn (P.Q.)

**Keywords:** SAW, reflective delay line, maximum phase measuring error, maximum range, maximum accuracy

## Abstract

In a surface acoustic wave (SAW) wireless sensor with a reflective delay line structure, three reflectors are often used to eliminate 2π ambiguity of phase measurement. The maximum range of the measured parameter and the maximum accuracy have recently been attracting much research attention. In this paper, an analytical formula for all the factors influencing the measurement range and accuracy of the delay line SAW sensor are deduced for the first time. The factors include: the sensor sensitivity, the topology of the delay line, the available wireless bandwidth and the allowed maximum phase measuring error of the reading system, which is easier to retrieve and more fully describes the possible noises than SNR. Additionally, many designers believe that increasing the reflector could improve accuracy continuously or realize multi-resolution measurement. However, they ignore some certain criteria that the reflector location must satisfy. The reachable maximum accuracy by every increase of a reflector is also presented. A SAW temperature sensor system using 128° YX-LiNbO_3_ is designed to verify the above theoretical analysis.

## 1. Introduction

The wireless passive sensor based on SAW technology features are: purely passive, harsh environment resistance and long service-life. Reflective delay line structure and resonator structure are two main transduction structures. The former is superior in terms of simplicity and easy combination with radio frequency identification technology. It has been widely used in applications in harsh environments [1,2,3,4,5,6].

As two important specifications, the measurement accuracy and the maximum range of the measurement have to be eclectically designed according to the practical applications. Here, please note that the range refers to the span over which the physical quantity is measured, rather than the maximum reading distance of the wireless reading system. It is known that the product of the time delay and the available wireless bandwidth limits the maximum information content retrievable from a reflective delay line sensor. However, the inevitable noises decrease the limitations in the practical reading system. In order to improve the sensor accuracy, the carrier phase of reflection echo, rather than the time delay is usually measured. All the uncertainty factors that may influence the phase measurement accuracy have been comprehensively discussed in [1]. Shmaliy *et al.* deduced the relationship between signal-to-noise-ratio (SNR) of reflection echo and phase difference measuring error through the maximum likelihood method [7]; Kuypers, Viikari and Stelzer *et al.* have discussed the influence of SNR of reflection echoes on measurement accuracy in the presence of additive white Gaussian noise (AWGN) [8,9,10]. According to the reference [1], the measured phase errors are not only just interfered by a Gaussian noise, but the phase noise errors from the local oscillators in the reading system, incoherent co-channel interference mainly caused by other industrial scientific and medical (ISM) band users, and so on. So the analysis of SNR taking only AWGN into account is not sufficient for determining the maximum measurement accuracy of wireless SAW sensors. Meanwhile, the limitation of the measurement range due to noises has not been considered until now. 

In this paper, an analytical formula for all the factors influencing the measurement range and accuracy of the delay line SAW sensor is deduced for the first time. The factors include: the sensor sensitivity, the topology of the delay line, the available wireless bandwidth and the allowed maximum phase measuring error of the reading system, which is easier to retrieve and more fully describes the possible noises than SNR. Additionally, many designers believe that increasing the reflector could improve accuracy continuously or realize multi-resolution measurement [11]. However, they ignore some certain criteria that the reflector location must satisfy. The reachable maximum accuracy by every increase of a reflector is also presented. Finally, a SAW temperature sensor system using 128° YX-LiNbO_3_ is designed to verify the above theoretical analysis. 

## 2. Principle of Sensing with Three Reflectors

**Figure 1:**
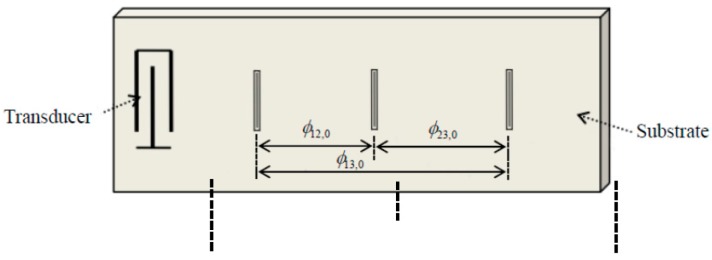
Structure of the delay-line sensor with three reflectors.

A SAW sensor with three reflectors is shown in Figure 1. Generally, the phase difference $\phi_{ij}$ between the *i*th reflector and the *j*th reflector is used to replace the time delay $\tau_{ij}$ in order to achieve high accuracy and eliminate effect of the Doppler shift and time varying channels. The mathematical expression is:

(1) $$\phi_{ij}=\phi_{j}-\phi_{i}\text{=2}\pi f_{c}(\tau_{j}-\tau_{i})=\text{2}\pi f_{c}\tau_{ij}(i,j=1,2,3,i\neq j)$$

When the measured value changes, the phase difference between reflectors will be different accordingly:

(2) $$\phi_{ij}=\phi_{ij,0}+\Delta \phi_{ij}=\phi_{ij,0}[1+S(D-D_{0})]=\phi_{ij,0}(1+S\cdot \Delta D)$$

(3) $$\Delta D=\frac{\Delta \phi_{ij}}{S\cdot \phi_{ij,0}}$$

where $\phi_{ij,0}$ is the phase difference under the referenced condition; $\Delta \phi_{ij}$ is the variation of phase difference; *D* is the current measured value; $D_{0}$ is the measured value under the referenced condition; ΔD is the variation of the measured value; and *S* is the sensor sensitivity, which is mainly determined by the substrate material for delay line structure.

The demodulated phase values from the reflective echoes are the decimal parts of the actual phase values. To eliminate phase ambiguity of 2π, a very short phase delay of $\phi_{23,0}-\;\phi_{12,0}$ has to be constructed so that the variation of phase difference $\Delta \phi_{23}-\Delta \phi_{12}$ will be less than 2π in the full measurement range [2,3].

Suppose $\delta_{\varphi \max}$ is the allowed maximum phase measuring error of the reading system. Note that it is different from the actually measured phase error $\delta_{\varphi }$, whose measurement technique and data manipulation using the measured data by a vector network analyzer or a frequency-stepped continuous wave reading system will described in Section 4. $\phi_{23,0}$ and $\phi_{12,0}$ are two random measuring errors, the allowed maximum measuring error of $\phi_{23,0}-\phi_{12,0}$ is $\sqrt{2}\delta_{\varphi \max}$. Theoretically, it is possible to construct a delay $\phi_{23,0}-c\cdot \phi_{12,0}(c>1)$ with a smaller interval to expand the measuring range [3], but the corresponding error will become at least $\sqrt{c^{2}+1}\cdot \delta_{\varphi \max}$. In the following, it can be seen that both the maximum measurement range and the accuracy decrease when *c* > 1. Therefore, *c =* 1 is adopted in this paper. 

Three different equations of the measured value could be concluded from Equation (3):

(4) $$\Delta D_{12}=\frac{\Delta \phi_{12}\pm \sqrt{2}\delta_{\varphi \max}}{S\cdot \phi_{12,0}}$$

(5) $$\Delta D_{13}=\frac{\Delta \phi_{13}\pm \sqrt{2}\delta_{\varphi \max}}{S\cdot \phi_{13,0}}$$

(6) $$\Delta D_{23-12}=\frac{\Delta \phi_{23}-\Delta \phi_{12}\pm 2\delta_{\varphi \max}}{S\cdot (\phi_{23,0}-\phi_{12,0})}$$

**Figure 2:**
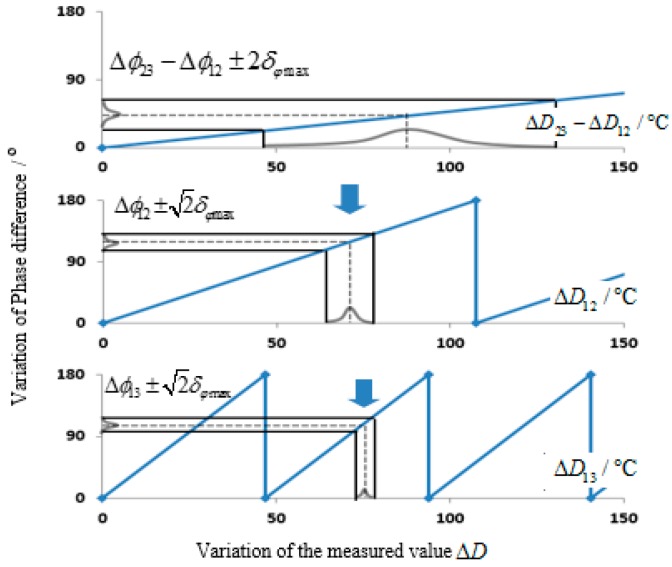
Determination process of the measured variation. (**a**) Curve of $\Delta \phi_{23}-\Delta \phi_{12}$; (**b**) Curve of $\Delta \phi_{12}$; (**c**) Curve of $\Delta \phi_{13}$.

Obviously, $\Delta D_{13}$ has the minimum measurement range, but the maximum measurement accuracy, because it has the maximum denominator $\phi_{13,0}$ and the minimum numerator $\Delta \phi_{13}$. On the contrary, $\Delta D_{23-12}$ has the maximum measurement range, but the minimum measurement accuracy. Figure 2 shows that although $\Delta \phi_{23}-\Delta \phi_{12}$ has low measurement accuracy, but it is adequate to judge the 2π period where $\Delta \phi_{12}$ belongs to. Similarly, the 2π integral part of $\Delta \phi_{13}$ could be judged by $\Delta \phi_{12}$. Combine it with the 2π decimal part represented by $\Delta \phi_{13}$ itself and the actual phase value could be gained. All normal distribution curves shown in Figure 2 are due to random measuring errors. This algorithm realizes the maximum measurement range of $\Delta D_{23-12}$ and the maximum measurement accuracy of $\Delta D_{13}$.

## 3. The Maximum Measurement Range and Accuracy

### 3.1. The Maximum Measurement Range

**Figure 3:**
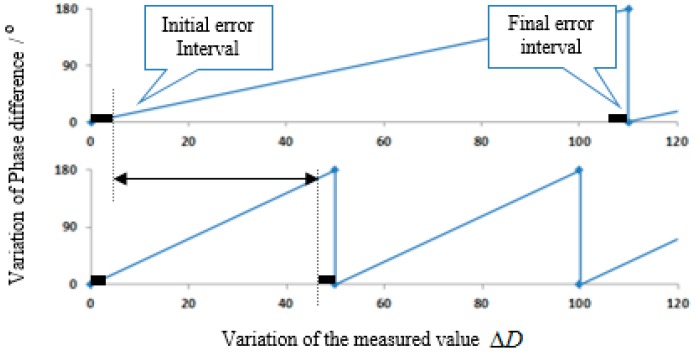
The initial error interval and the final error interval. (**a**) Curve of $\Delta \phi_{12}$; (**b**) Curve of $\Delta \phi_{13}$.

All signs and marks in this correspondence are subject to the one-way transmission length of SAW. In other words, the phase measurement range is 0~π and the corresponding measured variation range is 0~$R_{\max}$. Considering the phase measuring error of the system, the actual phase difference $\Delta \phi_{ij}=0$ and $\Delta \phi_{ij}=\pi$ corresponding to the measured variations $\Delta D_{ij}\neq 0$ and $\Delta D_{ij}\neq R_{\max}$, respectively. In this paper, uncertain ranges of the initial position and final position caused by the phase measuring error are called the initial error interval and the final error interval, which are shown in Figure 3 and Table 1.

**Table 1:** The initial error interval and the final error interval under various reference phase differences.

	Initial Error Interval	Final Error Interval
$\Delta D_{23-12}$	$0\sim \frac{4\delta_{\varphi \max}}{\phi_{23,0}-\phi_{12,0}}$	$\frac{\pi -4\delta_{\varphi \max}}{\phi_{23,0}-\phi_{12,0}}\sim \frac{\pi }{\phi_{23,0}-\phi_{12,0}}$
$\Delta D_{12}$	$0\sim \frac{2\sqrt{2}\delta_{\varphi \max}}{\phi_{12,0}}$	$\frac{\pi -2\sqrt{2}\delta_{\varphi \max}}{\phi_{12,0}}\sim \frac{\pi }{\phi_{12,0}}$
$\Delta D_{13}$	$0\sim \frac{2\sqrt{2}\delta_{\varphi \max}}{\phi_{13,0}}$	$\frac{\pi -2\sqrt{2}\delta_{\varphi \max}}{\phi_{13,0}}\sim \frac{\pi }{\phi_{13,0}}$

2π ambiguity could be unwrapped correctly as long as the above three groups of variations have an overlapped interval, that is:

(7) $$\frac{4\delta_{\varphi \max}}{\phi_{23,0}-\phi_{12,0}}<\frac{\pi -2\sqrt{2}\delta_{\varphi \max}}{\phi_{12,0}}$$

(8) $$\frac{2\sqrt{2}\delta_{\varphi \max}}{\phi_{12,0}}<\frac{\pi -2\sqrt{2}\delta_{\varphi \max}}{\phi_{13,0}}$$

Limited by manufacturing technique and the time delay resolution capability of the system, phase difference between any two reflectors shall be larger than the minimum phase difference $\phi_{\min}$. Then:

(9) $$\phi_{\min}=2\pi f_{c}\tau_{\min}=2\pi f_{c}\frac{1}{2B}=\frac{\pi f_{c}}{B}$$

(10) $$\phi_{12,0}=\mu \phi_{\min}=\frac{\mu \pi f_{c}}{B}$$

where $\tau_{\min}$ is the minimum time delay interval between reflectors; *B* is the interrogation bandwidth, the detectable minimum time delay interval between reflectors is 1/*B*, all signs and marks in this correspondence are subject to the one-way transmission length, so $\tau_{\min}=1/2B$; $f_{c}$ is the center frequency; and μ is the margin coefficient introduced in considering of time delay spread [1], which is usually larger than 1. Suppose $\phi_{23,0}=k\cdot \phi_{12,0}$ (*k* is the proportionality coefficient). Substitute it into Equations (3), (7) and (8):

(11) $$1+\frac{4\delta_{\varphi \max}}{\pi -2\sqrt{2}\delta_{\varphi \max}}<k<1+\frac{\pi }{S\cdot \phi_{12,0}\cdot R}$$

(12) $$k<\frac{\pi }{2\sqrt{2}\delta_{\varphi \max}}-2$$

To ensure that Equation (11) has solutions, it shall be:

(13) $$R<\frac{B(\pi -2\sqrt{2}\delta_{\varphi \max})}{4\delta_{\varphi \max}S\mu f_{c}}$$

Therefore, the maximum measurement range of the sensor is influenced by $\delta_{\varphi \max}$, *S*, *B*, $f_{c}$ and μ. Equation (13) reveals that to expand the measurement range as much as possible, μ shall be as small as possible in considering of delay spread, that is, $\phi_{12,0}$ shall reach its minimum. 

### 3.2. The Maximum Measurement Accuracy

According to Equation (5), the maximum measurement accuracy of using three reflectors is:

(14) $$\xi_{13}=\frac{\sqrt{2}\delta_{\varphi \max}}{S\cdot \phi_{13,0}}$$

It is well known that continuous increase of the number of reflectors could further improve the measurement accuracy. Suppose the 4th, 5th, …, (*m*−1)th, *m*th reflector are added if the substrate length is allowed and all reflection echoes have no significant amplitude differences, then the increasing reflector must be arranged reasonably to meet the 2π compensation rule of phase, *i.e.*, the maximum value of the initial error interval of $\Delta D_{1(m-1)}$ shall be smaller than the minimum value of the final error interval of $\Delta D_{1m}$:

(15) $$\phi_{1m,0}<\frac{\pi \phi_{1(m-1),0}}{2\sqrt{2}\delta_{\varphi \max}}-1\;(\text{m}=3,4,5\ldots )$$

Therefore, discussion on the measurement accuracy of the sensor is meaningful when Equation (15) is satisfied. Since $\phi_{1m,0}$ is limited by $\phi_{1(m-1),0}$, $\phi_{1(m-1),0}$ is also limited by $\phi_{1(m-2),0}$, and so on, the maximum measurement accuracy of the *m*th reflector is:

(16) $$\xi_{1m}={\left(\frac{\pi }{\sqrt{2}\delta_{\varphi \max}}\right)}^{m-3}\xi_{13}+\frac{2\sqrt{2}\delta_{\varphi \max}}{\pi -2\sqrt{2}\delta_{\varphi \max}}\left(1-{\left(\frac{\pi }{\sqrt{2}\delta_{\varphi \max}}\right)}^{m-3}\right)$$

## 4. Experimental Verification

**Figure 4:**
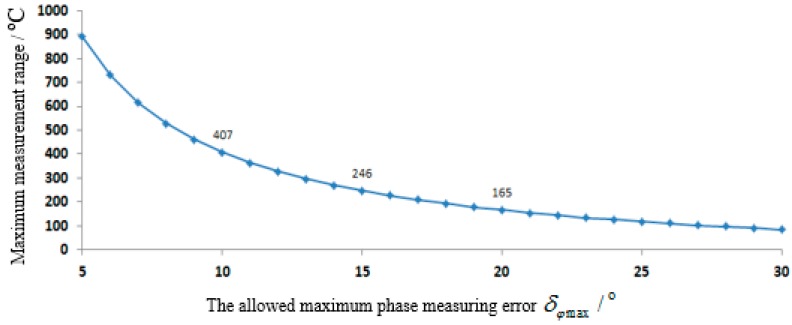
The relation curve between the maximum measurement range and the allowed maximum phase measuring error.

To verify the above analysis, a SAW temperature sensor system using 128° YX-LiNbO3 as the substrate is fabricated. The design parameters are *S* = −72 × 10^−6^/°C, $f_{c}$ = 922.5 MHz and *B* = 10 MHz. Therefore, the minimum time delay interval between two neighboring reflectors is $\tau_{\min}$ = 50 ns. Considering of time delay spread, the margin coefficient is μ = 1.4 and the reference time delay interval gained from Equation 10 is $\tau_{12,0}$ = 70 ns. Correspondingly, the relationship between $R_{\max}$ and $\delta_{\varphi \max}$ is shown in Figure 4. It can be seen that the maximum measurement range can be up to 407 °C when the measured maximum phase error of the reading system is within 10°.

The proportionality coefficient range of reflector according to Equations (11) and (12) is determined 1.2638<k<1.3585, $\tau_{23,0}$ = 90 ns is appropriate. The fourth reflector is added to further improve the measurement accuracy, in this case, $\tau_{14,0}$ = 320 ns and the measurement accuracy ξ = 1.85 °C.

### 4.1. VNA Test Results 

**Figure 5:**
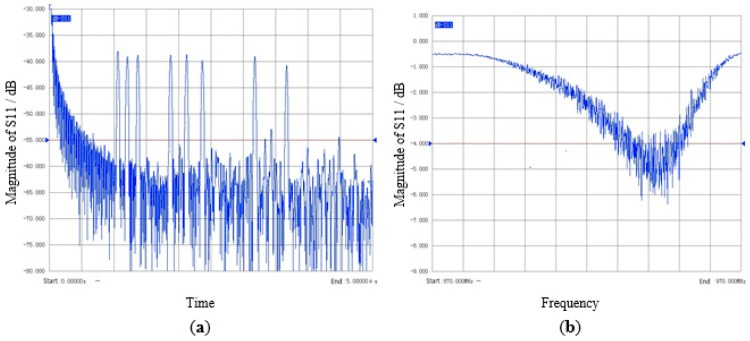
Measured S_11_ of a SAW tag prototype. (**a**) Time-domain response of surface acoustic wave (SAW) tag; (**b**) Frequency-domain response of SAW tag.

The fabricated SAW temperature sensor is put into a temperature control oven and the temperature variation ranges from 0 °C to 300 °C. The temperature sensing information is extracted by using a vector network analyzer (VNA, model: AV3629A) at 100 MHz bandwidth [3,8]. Time domain response and frequency domain response of the SAW tag tested by VNA is shown in Figure 5. 

**Figure 6:**
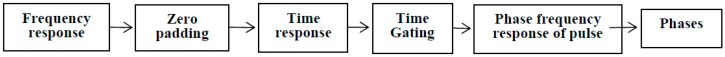
Phase calculation process through vector network analyzer (VNA).

As shown in Figure 6, we firstly use the frequency data 917.5 MHz~927.5 MHz to calculate the time response through the inverse Fourier Transform. In order to obtain enough fine time scale, zero-padding is implemented from 0 Hz to 1 GHz. Each pulse can be truncated by time-gating, *i.e.*, the points corresponding to an identified pulse are maintained while the rest of time response is set to a level close to zero. The FFT is then used to transform the truncated time-domain response back to the frequency domain. According to the phase frequency response, the phase value at the central frequency of each pulse can be achieved. The phase error $\delta_{\varphi }$ is less than 10° through this method, the requirement of $\delta_{\varphi }\;\leq \;\;\delta_{\varphi \max}$ is always satisfied. 

(17) $$\Delta D_{23-12}=\frac{1}{S\cdot (\phi_{23,0}-\phi_{12,0})}(\Delta \phi_{23}-\Delta \phi_{12}\pm 2\delta_{\varphi \max})$$

(18) $$\Delta D_{12}=\frac{1}{S\cdot \phi_{12,0}}(\Delta \phi_{12}\pm \sqrt{2}\delta_{\varphi \max}+180n_{12}),n_{12}=0,1,2...$$

(19) $$\Delta D_{13}=\frac{1}{S\cdot \phi_{13,0}}(\Delta \phi_{13}\pm \sqrt{2}\delta_{\varphi \max}+180n_{13}),n_{13}=0,1,2...$$

(20) $$\Delta D_{1k}=\frac{1}{S\cdot \phi_{1k,0}}(\Delta \phi_{1k}\pm \sqrt{2}\delta_{\varphi \max}+180n_{1k}),n_{1k}=0,1,2...$$

Theoretically, there exists an unique overlap range between $\Delta D_{23-12}$, $\Delta D_{12}$,…, $\Delta D_{1k}$, and $n_{12}$, $n_{12}$,…, $n_{12}$ are the number of 2π period for compensation respectively. $\Delta D_{1k}$ is the final result we need.

**Figure 7:**
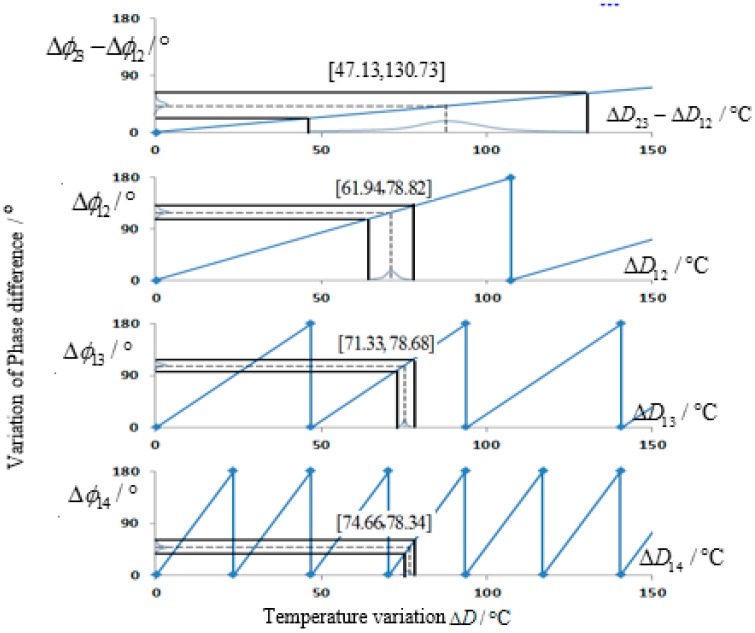
Determination process of temperature variation.

The detailed process of phase compensation is shown in Figure 7. Take an example, when the temperature in the oven *D* = 110 °C, calculation results come from VNA are $\Delta \phi_{23}-\Delta \phi_{12}=42.55{}^{\circ}$, $\Delta \phi_{12}=117.89{}^{\circ}$, $\Delta \phi_{13}=108.09{}^{\circ}$ and $\Delta \phi_{14}=48.46{}^{\circ}$. Since the error of $\Delta \phi_{23}-\Delta \phi_{12}$ is $2\delta_{\varphi \max}=20{}^{\circ}$, the possible temperature corresponding to the phase difference interval [22.55, 62.55] ranges from 47.13 °C to 130.73 °C. Similarly, to Error of $\Delta \phi_{12}$ is $\sqrt{2}\delta_{\varphi \max}=14.14{}^{\circ}$ and the temperature variation range under the phase difference interval [103.75, 132.03] is [61.94 + 107.5n, 78.82 + 107.5n], n = 0, 1, 2, 3… Only [61.94, 78.82] overlaps with the temperature variation range $\Delta \phi_{23}–\Delta \phi_{12}$, that is, the actual phase values $\Delta \phi_{12}=117.89{}^{\circ}$. Similarly, only [71.33, 78.68] in $\Delta \phi_{13}$ overlaps with the temperature variation range of $\Delta \phi_{12}$. Therefore, it needs compensation of a 2π period. Actually, $\Delta \phi_{13}=108.09{}^{\circ}+180{}^{\circ}=288.09{}^{\circ}$. In $\Delta \phi_{14}$, only [74.66, 78.34] overlaps with the temperature variation range of $\Delta \phi_{13}$, thus needing to compensate three 2π periods. Actually, $\Delta \phi_{14}=48.46{}^{\circ}+3\times 180{}^{\circ}=588.46{}^{\circ}$.

Based on the above steps, phase ambiguity of $\Delta \phi_{13}$ and $\Delta \phi_{14}$ is eliminated and the intermediate value of [74.66, 78.34] as the final result, that is, $\Delta D_{13}=76.5\;{}^{\circ}\text{C}$. The actual temperature variation is $\Delta D_{real}=D-D_{0}=77\;{}^{\circ}\text{C}$ and the error is ξ=0.5°, which agrees with the design accuracy. 

**Figure 8:**
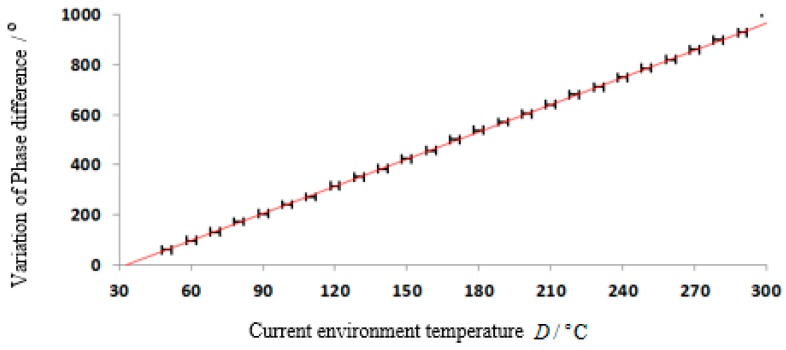
Relation between $\Delta \phi_{13}$ and D in the VNA test.

Figure 8 is the comparison between the measured value and the theoretical value. The black mark is the measured phase difference through phase compensation and the straight line is the theoretical value. The designed SAW temperature sensor could meet requirements of measurement range and accuracy. 

### 4.2. Reader Test Results

**Figure 9:**
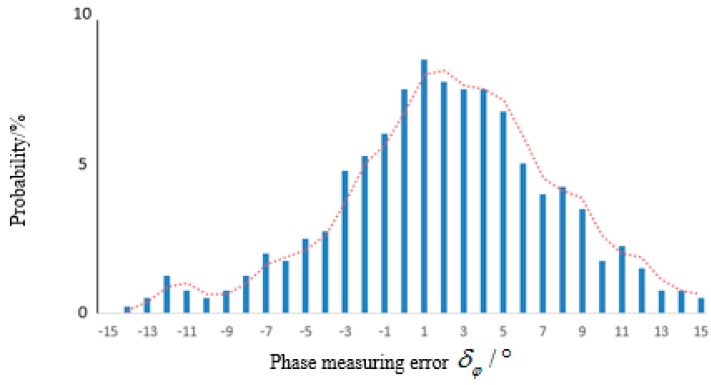
Distribution of phase measuring error.

**Figure 10:**
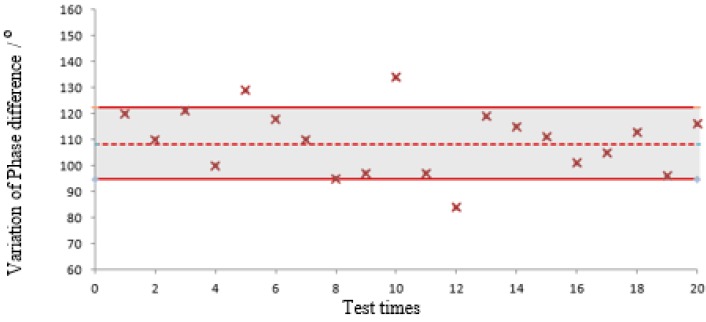
Multiple measurement results of $\Delta \phi_{13}$ in the reader test.

In this experiment, the SAW sensor is interrogated by a self-developed reader with the 10 MHz bandwidth, and the sensor and the antenna of the reader are deliberately isolated by the metal door of the temperature control oven (type: LP-GDS-100), so as to keep a very large phase measuring error. Compared to the VNA test above, the actual phase measuring error $\delta_{\varphi }$ in this experiment is much larger, even exceeds the threshold $\delta_{\varphi \max}$. The distribution of phase error is shown in Figure 9, which exceeds 10° more than 300 times among 3000 samples. The measured results under 110 °C are shown in Figure 10, the theoretical value $\Delta \phi_{13}=108.09{}^{\circ}$ is represented by dashed line, the numerical range $\Delta \phi_{13}\pm \sqrt{2}\delta_{\varphi \max}$ = (94.76, 122.23), upper and lower bounds are represented by solid lines. It can be seen that $\Delta \phi_{13}$ fluctuate violently and some of them (the 5th, 8th, 10th, 12th) exceed the design accuracy.

## 5. Conclusions

In this paper, the threshold $\delta_{\varphi \max}$ covers all possible noises, including Gaussian noise, phase noise errors from the local oscillators, incoherent co-channel interference, *etc*., which is easier to retrieve and more fully describes the possible noises than SNR. On this basis, the maximum measurement range and accuracy of SAW sensor with a delay line structure are introduced. Furthermore, how to locate every increase of a reflector to realize the maximum measurement accuracy were deduced for the first time. A SAW temperature sensor system using 128° YX-LiNbO_3_ is designed to verify the research results. The conclusion is also applicable to the temperature compensation of SAW RFID with phase encoding using a minimum number of reference reflectors [12]. 
